# Nucleotide Excision Repair and Transcription-coupled DNA Repair Abrogate the Impact of DNA Damage on Transcription*

**DOI:** 10.1074/jbc.M115.685271

**Published:** 2015-11-11

**Authors:** Aditi Nadkarni, John A. Burns, Alberto Gandolfi, Moinuddin A. Chowdhury, Laura Cartularo, Christian Berens, Nicholas E. Geacintov, David A. Scicchitano

**Affiliations:** From the ‡ Departments of Biology and Chemistry, New York University, New York, New York 10003,; the § Dipartimento di Matematica e Informatica “Ulisse Dini,” Università di Firenze, 50134 Firenze, Italy,; the ‖ Institute of Molecular Pathogenesis, Friedrich-Loeffler-Institut, Jena, Germany, 07743, and; the ¶ Division of Science, New York University Abu Dhabi, Post Office Box 129188, Abu Dhabi, United Arab Emirates

**Keywords:** carcinogenesis, DNA repair, nucleotide excision repair, RNA polymerase II, transcription

## Abstract

DNA adducts derived from carcinogenic polycyclic aromatic hydrocarbons like benzo[*a*]pyrene (B[*a*]P) and benzo[*c*]phenanthrene (B[*c*]Ph) impede replication and transcription, resulting in aberrant cell division and gene expression. Global nucleotide excision repair (NER) and transcription-coupled DNA repair (TCR) are among the DNA repair pathways that evolved to maintain genome integrity by removing DNA damage. The interplay between global NER and TCR in repairing the polycyclic aromatic hydrocarbon-derived DNA adducts (+)-*trans-anti*-B[*a*]P-*N*^6^-dA, which is subject to NER and blocks transcription *in vitro*, and (+)-*trans-anti*-B[*c*]Ph-*N*^6^-dA, which is a poor substrate for NER but also blocks transcription *in vitro*, was tested. The results show that both adducts inhibit transcription in human cells that lack both NER and TCR. The (+)-*trans-anti*-B[*a*]P-*N*^6^-dA lesion exhibited no detectable effect on transcription in cells proficient in NER but lacking TCR, indicating that NER can remove the lesion in the absence of TCR, which is consistent with *in vitro* data. In primary human cells lacking NER, (+)-*trans-anti*-B[*a*]P-*N*^6^-dA exhibited a deleterious effect on transcription that was less severe than in cells lacking both pathways, suggesting that TCR can repair the adduct but not as effectively as global NER. In contrast, (+)-*trans-anti*-B[*c*]Ph-*N*^6^-dA dramatically reduces transcript production in cells proficient in global NER but lacking TCR, indicating that TCR is necessary for the removal of this adduct, which is consistent with *in vitro* data showing that it is a poor substrate for NER. Hence, both global NER and TCR enhance the recovery of gene expression following DNA damage, and TCR plays an important role in removing DNA damage that is refractory to NER.

## Introduction

Chemicals and radiation can damage DNA, resulting in structural alterations to the nitrogenous bases, deletions of genetic material, and breaks in chromosomes (1–4). The resulting damage often disrupts fundamental cellular processes, including replication and transcription. During replication, DNA damage interferes with DNA synthesis, introduces genetic errors, and increases genomic instability, events often associated with cancer (5, 6). During transcription, damaged DNA can disrupt RNA synthesis, resulting in truncated transcripts, or introduce errors into the products during elongation (7).

Although the effects of DNA damage on replication have been well characterized, less is known about its influence on gene expression in cells. There is considerable biochemical evidence that DNA damage profoundly affects the progression of RNA polymerases during RNA synthesis (8). A DNA lesion situated on the transcribed strand often stalls or strongly impedes RNA polymerase translocation, resulting in altered transcripts that can be truncated, contain deletions or insertions, or harbor misincorporated bases (9–11). In fact, alterations to mRNA generated during transcription, an event referred to as transcriptional mutagenesis, can result in changes to protein function following translation (12). For example, *O*^6^-methylguanine, a DNA lesion formed by methylating agents, partially blocks transcription elongation of human RNA polymerase and frequently directs insertion of a uridine nucleotide rather than a cytidine nucleotide into RNA (13, 14).

To minimize the adverse consequences of DNA damage, genome maintenance pathways have evolved in cells (15–17). Among them is nucleotide excision repair (NER)^3^ that has two branches, global NER and transcription-coupled DNA repair (TCR), each of which is germane to the work reported here.

Global NER is executed via a complex set of coordinated events. The pathway requires over 30 proteins, including the XPA through XPG proteins, that are defective in patients with the disease xeroderma pigmentosum (XP). The first step in global NER, which distinguishes it from TCR, is DNA damage recognition that is accomplished in part by a protein complex in human cells called XPC/hR23B. The heterodimer binds to chemically modified bases in DNA that interrupt base pairing and distort the double helix, thus allowing global NER to remove a wide array of lesions from DNA (18, 19). Following damage recognition, the additional NER proteins orchestrate the removal of the damaged base as part of a DNA oligomer that is typically 30–35 bases in length in eukaryotes, generating a single-stranded gap in the original vicinity of the damage (17, 20). Subsequent synthesis and ligation of nascent DNA fill the gap and complete the repair process (18, 21).

TCR removes DNA damage from the transcription units of actively expressed genes; hence, this pathway, unlike global NER, operates on very specific regions of the genome (22, 23). TCR requires many of the NER XP proteins, with the exception of XPC. In addition, TCR needs active CSA and CSB proteins that are defective in patients with Cockayne syndrome (CS) (24). During TCR, an elongating RNA polymerase detects DNA damage, presumably by stalling at lesions within the transcription unit, obviating the need for XPC/hR23B. The stalled transcription complex, along with CSA, CSB, XPA, and other proteins, stimulates removal of the DNA adduct and repair of the gene's transcription unit (9, 25, 26).

**FIGURE 1.**
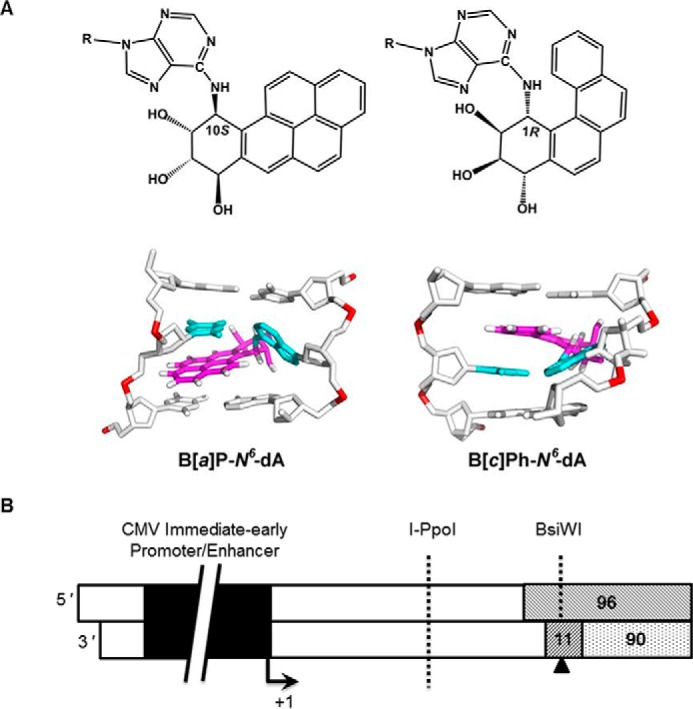
**The structures of B[*a*]P-*N*^6^-dA and B[*c*]Ph-*N*^6^-dA and the site-specific modified DNA for *in vitro* transcription are illustrated.** *A*, the B[*a*]P-*N*^6^-dA adduct disrupts base pairing at sites where it occurs in DNA, significantly distorting the double helix. In contrast, the B[*c*]Ph-*N*^6^-dA adduct does not disrupt base pairing at sites where it occurs in DNA. Both adducts are intercalated between base pairs, but the B[*a*]P-*N*^6^-dA adduct, which is planar, rigid, and more bulky, causes disruption of Watson-Crick base pairing, whereas the smaller, curved B[*c*]Ph ring system stacks between base pairs without rupturing the Watson-Crick pairs. *B*, templates for *in vitro* transcription contained the CMV immediate early promoter/enhancer, as shown schematically in *black*. The +1 start site for transcription is indicated, and the *arrow* designates the direction of transcription elongation. The region formed by annealing the 96-mer, the 90-mer, and the 11-mer is shown. The position of the B[*a*]P-*N*^6^-dA adduct within the 11-mer is indicated with a *triangle* on the transcribed strand. In the unmodified, control DNA template, the position indicated by the *triangle* contained dA. The size of the template was 1,141 bp following isolation from the paramagnetic beads. The recognition sites for I-PpoI and BsiWI are also shown.

The existence of multiple ways to detect DNA damage may well play an important role in removing DNA adducts that are identified efficiently by one pathway and poorly by another. Consider, for example, DNA adducts formed following exposure to polycyclic aromatic hydrocarbons (PAHs) (27). PAHs arise as ubiquitous byproducts of incomplete combustion and include benzo[*a*]pyrene (B[*a*]P) and benzo[*c*]phenanthrene (B[*c*]Ph), both of which are known procarcinogens in humans (28–30). Certain cells, particularly hepatocytes, metabolize the procarcinogenic PAHs to diol epoxide intermediates, which in turn react with DNA, primarily at purines, producing stereoisomeric *trans* and *cis* adducts. Among the adducts produced by B[*a*]P-diol epoxide and B[*c*]Ph-diol epoxide are (+)-*trans-anti*-B[*a*]P-*N*^6^-dA (B[*a*]P-*N*^6^-dA) and (+)-*trans-anti*-B[*c*]Ph-*N*^6^-dA (B[*c*]Ph-*N*^6^-dA) (Fig. 1*A*) (31). Structural studies show that these two adducts exhibit distinct conformational states (31–33). The B[*a*]P-*N*^6^-dA adduct disrupts base pairing, resulting in the induction of major distortions within the DNA helix; the B[*c*]Ph-*N*^6^-dA adduct does not disrupt base pairing, but it does induce local unwinding and stretching of the double helix to accommodate the adduct. Indeed, biochemical assays show that B[*a*]P-*N*^6^-dA adducts are subject to removal by global NER, whereas B[*c*]Ph-*N*^6^-dA adducts are resistant to global NER (34). These striking differences have been analyzed at the molecular level, but it is unknown whether or not they manifest themselves at the cellular level.

An obvious implication of the existence of TCR is that DNA adducts that are typically refractory to global NER but pose blocks to transcription could be subject to TCR. The work reported here examined the effects of B[*a*]P-*N*^6^-dA and B[*c*]Ph-*N*^6^-dA adducts on transcription in primary human fibroblasts that are either DNA repair-proficient or lack global NER, TCR, or both. Either B[*a*]P-*N*^6^-dA or B[*c*]Ph-*N*^6^-dA was positioned on the transcribed strand of a reporter gene that encodes red fluorescent protein (RFP), specifically within the 5′-UTR of the gene's transcription unit. Human fibroblasts were transfected with the site-specifically modified DNA or an unmodified control. The quantity of RFP mRNA and the production of functional RFP were monitored over time. The results obtained for the site-specifically modified DNA were then compared with those of the corresponding, unmodified control DNA.

The data indicate that DNA adducts that pose strong blocks to transcription interfere with gene expression in cells. Furthermore, a cell's inability to remove the damage results in very slow, relatively poor recovery of transcription with an associated limited production of functional protein. Both global NER and TCR enhance the recovery of gene expression compromised by a DNA adduct on the transcribed strand, with TCR playing a particularly important role in recovery from a lesion that is refractory to recognition and removal by global NER.

## Experimental Procedures

### 

#### 

##### Chemicals, Reagents, and Cell Culture Materials

Chemicals and other reagents were obtained from Thermo Fisher Scientific and Sigma-Aldrich. Radioactive isotopes were acquired from PerkinElmer Life Sciences (Waltham, MA). The IDT 20/100 ladder, PCR primers, and fluorescent hydrolysis probes were obtained from Integrated DNA Technologies (Coralville, IA), and oligomers for transcription template preparation, plasmid modification, and vector synthesis were from Sigma-Aldrich. The 50-bp DNA ladder and enzymes other than those indicated below were obtained from New England Biolabs (Ipswich, MA).

##### In Vitro Transcription

Templates suitable for *in vitro* transcription were prepared as described (35). In brief, plasmid pCI-neo-G-less-T7, which contains the CMV immediate early promoter/enhancer element that supports human RNA polymerase II (hRNAPII) transcription and lacks eukaryotic origins of replication, was cut with restriction enzyme BbsI (New England Biolabs). The restriction site was annealed to a set of oligomers: an 11-mer, a 96-mer, and a 90-mer containing a 5′-biotin tag (Table 1). The 11-mer 5′-CTCGTACGCTC-3′ was either unmodified at the sole adenine or modified with a site-specific B[*a*]P-*N*^6^-dA inserted in place of adenine, and each was used to assemble a control or damaged template, respectively (Fig. 1*B*). T4 DNA ligase (Bayou Biolabs, Metairie, LA) was added along with 1 mm ATP, and the mixture was incubated for 16 h at 16 °C. The product was precipitated using Streptavidin MagneSphere® paramagnetic particles (Promega Corp., Madison, WI) that bound to the biotin tag. The product bound to the paramagnetic particles was digested with BglII (New England Biolabs) to remove all elements of pCI-neo-G-less-T7 not necessary for *in vitro* transcription. The template was then removed from the paramagnetic particles by digestion with EcoRV (New England Biolabs), and the resulting DNA was purified using 1% agarose gel electrophoresis in 89 mm Tris, 89 mm borate, 2 mm Na_2_EDTA (pH 8.3 at 25 °C) followed by extraction from the gel using the QIAQuick gel extraction kit (Qiagen, Valencia, CA). Finally, the templates were tested for the absence of nicks and the presence of the B[*a*]P-*N*^6^-dA lesion (35).

**TABLE 1:** **Primers for *in vitro* template construction** The position of the B[*a*]P-*N*^6^-dA adduct in the 11-mer is indicated with an underlined “A” in boldface type.

Oligomer	Sequence
96-mer	5′-TTGCGAGCGTACGAGGTGCTGTACTCAGGTGTGGAATCAACCCACAGCTGACAGGGCAGGTCTTGGCCAGTTGGGATATCCAAAACATCTTGTTGA-3′
90-mer	5′-Biotin-TTTTTTTTTTCAACAAGATGTTTTGGATATCCCAACTGGCCAAGACCTGCCCTGTCAGCTGTGGGTTGATTCCACACCTGAGTACAGCAC-3′
11-mer	5′-CTCGT**A**CGCTC-3′

*In vitro* transcription reactions were performed using the HeLaScribe® nuclear extract *in vitro* transcription system (Promega) as the source of hRNAPII and other essential transcription factors (35, 36). In brief, reactions were carried out in a 25-μl volume with 50 fmol of template in transcription buffer (20 mm HEPES (pH 7.9), 100 mm KCl, 0.2 mm EDTA, 0.5 mm DTT, 20% glycerol), 400 μm ATP, 400 μm GTP, 400 μm UTP, 16 μm [α-^32^P]CTP (∼25 Ci/mmol), and 8 units of HeLa nuclear extract. The mixture was incubated at 30 °C and quenched at an appropriate time with HeLaScribe® kit stop solution (0.3 m Tris-HCl (pH 7.4 at 25 °C), 0.3 m sodium acetate, 0.5% SDS, 2 mm EDTA, 3 μg/ml tRNA). RNA was isolated by extraction with phenol/chloroform/isoamyl alcohol (25:24:1, v/v/v) followed by ethanol precipitation. The RNA was resuspended in nuclease-free water, mixed with an equal volume of loading dye (98% formamide, 10 mm Na_2_EDTA, 0.1% xylene cyanol, 0.1% bromphenol blue), denatured at 90 °C for 10 min, and resolved with 7% denaturing PAGE using 8 m urea at 2,000 V for ∼3.5 h. The gel was dried and exposed to a BAS-IP MS 2040 E multipurpose standard storage phosphor screen (GE Healthcare). The screen was scanned using an FLA Typhoon 9000 Imager (GE Healthcare Life Sciences). The transcripts were quantified using band densitometry analysis in Fiji (37).

##### Vector Synthesis for Transcription Studies in Cells

**FIGURE 2.**
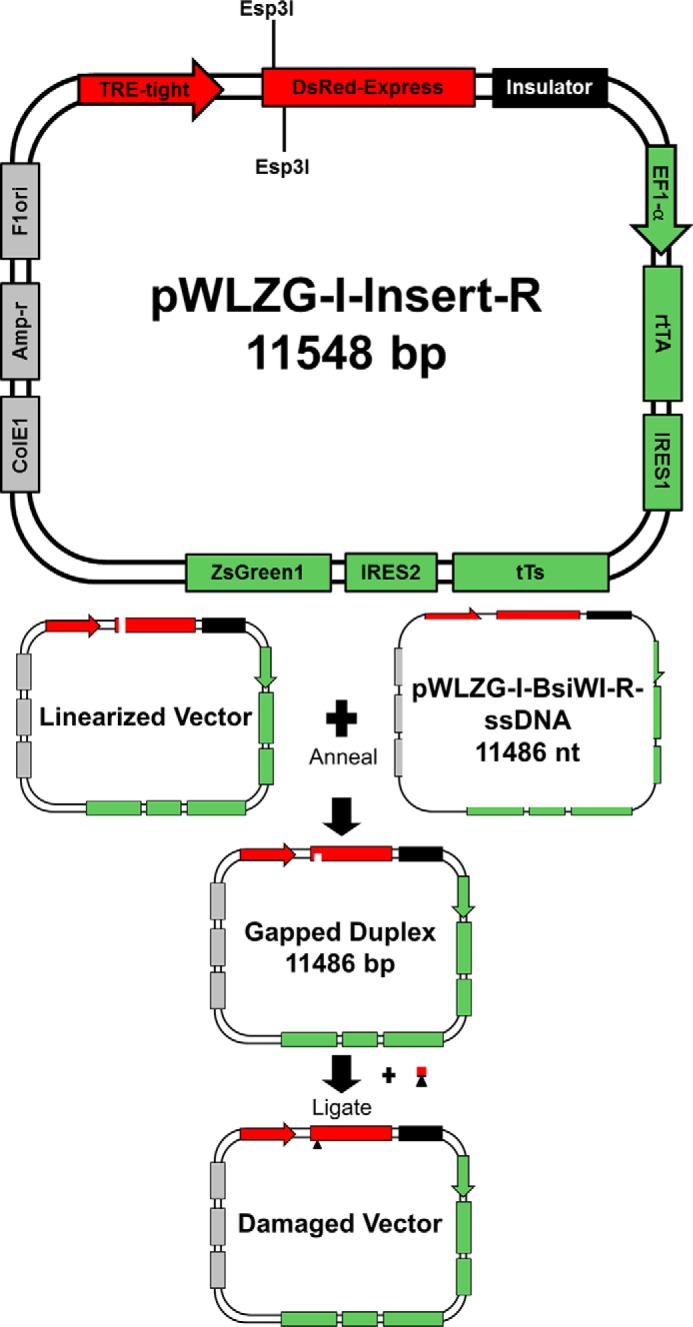
**Schematic for the gapped duplex system to assemble the site-specifically modified vectors for transcription analysis in cells.** The map of the parent vector for this work, pWLZG-I-Insert-R, is shown in detail and is divided into three functional regions. Region I, which is encoded in *shades of green*, has the following elements: the constitutive elongation factor 1-α (*EF1-*α) promoter, which drives transcription of the region following it to generate a polycistronic mRNA that encodes three proteins, each separated by an internal ribosomal entry sequence (*IRES*). The three encoded proteins include: tetR-derived reverse transactivator protein (*rtTA*), tetR-derived transrepressor protein (*tTS*), and GFP (*ZsGreen1*). IRES1 is derived from the polio virus genome, and IRES2 is derived from the encephalomyocarditis virus genome. Region II, which is encoded in *shades of red*, has the following elements: DsRed-express, which encodes RFP, and a tetracycline-responsive promoter element (*TRE-tight*) that drives Ds-Red-express transcription. Region III, which is encoded in shades of *gray*, contains components needed to propagate the plasmid in bacteria and prepare single-stranded DNA: an *E. coli* origin of replication (*ColE1*), an ampicillin resistance gene (*Amp-r*), and the F1 phage origin of replication (*F1ori*). Following transfection with the control or damaged vector, expression of the polycistronic mRNA in Region I produces transrepressor protein that binds to the tetracycline-responsive promoter element, repressing transcription of the RFP gene. The reverse transactivator protein and GFP are also expressed. After the addition of doxycycline to the transfected cells, the drug binds to transrepressor protein, which releases it from the tetracycline-responsive promoter element, thus removing the repressor; in concert, doxycycline binds to the reverse transactivator protein, creating a potent activator that binds to the tetracycline-responsive promoter element, permitting transcription of the RFP gene. Note that a chicken HS4 insulator element (*Insulator*), which is encoded in *black*, is positioned between Regions I and II to separate the two transcription units. The oligomer that ligated into the gapped duplex DNA is illustrated with a *red line*; the *black triangle* represents the presence of a site-specific DNA adduct.

Site-specific, modified vectors were synthesized by using a gapped duplex method (Fig. 2) that involved the preparation of single-stranded, closed circular DNA (38–40). In brief, closed circular, single-stranded DNA corresponding to the non-transcribed strand of the RFP gene in vector pWLZG-I-BsiWI-R was prepared using M13 bacteriophage (41). *E. coli* strain MV1190 (American Type Culture Collection, Manassas, VA) was transformed with plasmid WLZG-I-BsiWI-R. Log phase cultures of the transformed bacteria were superinfected with M13 helper phage VCSM13 (Agilent Technologies, Inc., Santa Clara, CA) at a multiplicity of infection greater than 10:1 phage/bacteria. Infected cultures were grown overnight at 37 °C in 2× YT medium (16 g/liter Bacto Tryptone, 10 g/liter yeast extract, 86 mm NaCl (pH 7.0)). Bacteria were pelleted, and bacteriophage that contained single-stranded DNA were recovered by polyethylene glycol precipitation. Single-stranded, circular DNA was recovered from helper phage by phenol extraction.

A modified oscillating phenol reassociation technique was used to generate gapped duplex DNA (42). In brief, the double-stranded DNA vector pWLZG-I-Insert-R was linearized by digestion with Esp3I (250 units/mg plasmid) in Tango Buffer (Thermo Fisher Scientific) with 1 mm DTT for 4 h at 37 °C. Linear pWLZG-I-Insert-R was mixed with single-stranded, closed circular WLZG-I-BsiWI-R at a molar ratio of 1:5 to form the gapped duplex. The DNA mixture was denatured by the addition of 1 m NaOH to a final concentration of 0.3 m and incubated at room temperature for 15 min. The mixture was neutralized with 3 m MOPS free acid to reach a final concentration of 0.4 m MOPS. NaCl and EDTA were added to final concentrations of 0.75 m and 1 mm, respectively, and buffered phenol was added to 10% of the final volume. The reaction was mixed rapidly with a micropipette to form a phenol emulsion that was cycled between 0 and 65 °C at 90 s/cycle for 45 min to foster association between the transcribed strand of linear pWLZG-I-Insert-R and the single-stranded, closed circular WLZG-BsiWI-R. This resulted in the formation of duplex DNA with an 11-nucleotide gap on the transcribed strand of the vector. The gapped duplex DNA was purified by eliminating excess single-stranded DNA using benzoylated naphthoylated DEAE-cellulose (Sigma-Aldrich) followed by phenol/chloroform extraction (43).

A DNA oligomer 5′-CTCGTACGCTC-3′ containing either B[*a*]P-*N*^6^-dA or B[*c*]Ph-*N*^6^-dA (36, 44–47) was phosphorylated using T4 polynucleotide kinase (New England Biolabs) in the presence of 1 mm ATP. The resulting oligomer was ligated into the gapped duplex using a 3:1 molar ratio of oligomer to gapped duplex DNA for 16 h at 16 °C in the presence of T4 DNA ligase (Bayou Biolabs) and 1 mm ATP. The mixture was extracted three times with 1% Triton X-114 equilibrated with 10 mm Tris-HCl, 1 mm Na_2_EDTA (pH 7.8 at 25 °C) (TE), precipitated by centrifugation, and resuspended in TE. The resulting DNA was purified by CsCl gradient ultracentrifugation (1.01 g/ml water) in the presence of ethidium bromide (0.4 mg/ml). The final product, WLZG-I-B[*a*]P-R or WLZG-I-B[*c*]Ph-R, was a covalently closed circular plasmid containing the DNA sequence of the control vector (WLZG-I-BsiWI-R) with a single modified base. The presence of the damaged base was verified by resistance to digestion with restriction endonucleases (Thermo Fisher Scientific).

##### Cell Culture and Transfection

Cell culture medium and cell culture vessels were acquired from Corning Inc. PBS was purchased from Fisher. Fetal bovine serum (FBS) was obtained from Atlanta Biologicals (Norcross, GA). Penicillin, streptomycin, and cell culture supplies were purchased from Sigma-Aldrich and Thermo Fisher Scientific. Primary fibroblasts (Coriell Cell Repositories, Camden, NJ) were grown in minimal essential medium supplemented with 15% (v/v) tetracycline system-approved FBS, 1 mm sodium pyruvate, 100 units/ml penicillin, and 100 μg/ml streptomycin. The cells were maintained in a humidified incubator at 37 °C under 5% CO_2_. Cells were transiently transfected using Transit-2020 transfection reagent (Mirus Bio LLC, Madison, WI) according to the manufacturer's instructions. In brief, cells were seeded 24 h prior to transfection at a density of 60,000 cells/well, where each well was 3.8 cm^2^ in area. Each plasmid was diluted in a mixture of Opti-MEM I reduced serum medium (Life Technologies, Inc.) to a final concentration of 5 ng/μl. The Transit 2020 transfection reagent was added to the diluted DNA mixture at a ratio of 2:1, with 1 μl of the reagent per 500 ng of DNA. The mixture was incubated at room temperature for 30 min and then added to the cells in each well for a final plasmid concentration of 0.5 ng/μl. (This corresponded to a final plasmid concentration of 7 × 10^−5^ pmol/μl.) The cells were incubated at 37 °C in humidified 5% CO_2_ for 24 h, after which the transfection reagent was removed, the cells were washed with PBS, and fresh minimal essential medium containing 15% FBS and 1 μg/ml doxycycline was added to induce transcription of the RFP gene. The point immediately prior to induction was denoted as time 0.

##### mRNA Quantitation

Following induction of transcription by the addition of doxycycline, cell lysates were collected at various time points, and RNA was isolated using the RNeasy minikit (Qiagen). In brief, the medium was removed, and the cells were immediately lysed by the addition of 350 μl of Buffer RLT (Qiagen) containing 10 μm β-mercaptoethanol/well. The lysates were placed on dry ice and then stored at −80 °C. The samples were thawed at 4 °C, and RNA was extracted from the lysates with the RNeasy Mini Kit following the manufacturer's protocol. Polyadenylated mRNA was converted to cDNA using an RT primer containing an anchored poly(dT) region that bound to the 5′-end of the poly(A) tail of polyadenylated mRNA, thus acting as a primer for RT while simultaneously adding a unique, 5′-end for subsequent amplification via PCR (Table 2). The AffinityScript multiple temperature cDNA synthesis kit (Agilent Technologies, Inc.) was used for cDNA synthesis according to the manufacturer's instructions. In brief, isolated RNA was mixed with the RT primer, incubated at 65 °C for 5 min, and slowly cooled to 4 °C to permit annealing. The RT reaction was carried out in 50 mm Tris-HCl (pH 8.3), 75 mm KCl, 3 mm MgCl_2_, 10 mm DTT, and 4 mm dNTPs in the presence of RT enzyme. The samples were incubated for 10 min at 25 °C followed by 1 h at 42 °C.

**TABLE 2:** **Primers for qRT-PCR-mediated quantification of RFP and GFP mRNA**

Oligomer	Sequence
Anchored poly(dT) RT primer	5′-ATGTTGACGCAGCCAGTGACT_20_VN-3′*^a^*
GFP-cDNA-FWD	5′-TAGTTGCCAGCCATCTGTTG-3′
GFP-cDNA-PROBE	5′-FAM-AAGGTGCCA/ZEN/CTCCCACTGTCCTTT-IBFQ-3′*^b^*
cDNA-REV	5′-ATGTTGACGCAGCCAGTGAC-3′
RFP-cDNA-FWD	5′-GAGGCTAACTGAAACACGGA-3′
RFP-cDNA-PROBE	5′-FAM-AGGAGACAA/ZEN/TACCGGAAGGAACCC-IBFQ-3′*^b^*

*^a^* V represents A, C, or G; N represents A, C, G, or T.

*^b^* FAM, 6-FAM^TM^; ZEN, ZEN^TM^ internal quencher; IBFQ, Iowa Black® FQ.

RFP cDNA and GFP cDNA were quantified by quantitative RT-PCR in the presence of fluorescent hydrolysis probes containing ZEN^TM^ and Iowa Black® FQ quenchers (Table 2). The cDNA-FWD primer complementary to the 3′-region of the cDNA generated from the RT reaction was added along with cDNA-REV primer, which was identical to the unique 5′-region of the RT primer. qPCRs were carried out using GoTaq Hot Start Colorless Master Mix (Promega) in a MyiQ^TM^ single-color real-time PCR detection system run with MyiQ Optical System Software version 2.0 (Bio-Rad). qPCRs were completed in duplicate. Baseline-subtracted raw fluorescence measurements were converted into a text file suitable for an R programming environment (48), and the data were processed with the qpcR module. qPCR curves were fit using the modlist function of qpcR (49). The Cy0 value (50) for each curve was obtained using the Cy0 function in qpcR, and the mean Cy0 (*Cy*0) for each was calculated. The average efficiency value for each amplicon (*Eff*_RFP_ and *Eff*_GFP_) on each plate was calculated by computing the slope of the line through the log_2_ of the fluorescence value at the cycle nearest the Cy0 value and the two prior and two latter cycles for each reaction on a plate and then determining the mean efficiency of each amplicon (51, 52). The final value for each experimental measurement was calculated as (*Eff*_RFP_)^−^^Cy0^ and (*Eff*_GFP_)^−^^Cy0^ for each RFP and GFP measurement. The procedure is similar to the LinRegPCR method of efficiency correction in using an average efficiency per plate (51).

Statistical parameters were calculated using RFP/GFP-normalized data as follows. At least three independent measurements were made for each experimental condition, with an experimental condition defined as a unique time point, cell line, and DNA template. Each measurement had one RFP and one GFP value, with one RFP or GFP value calculated as the (*Eff*_RFP_)^−^*^Cy0^* and (*Eff*_GFP_)^−^*^Cy0^* value for that measurement. The total number of measurements per experimental condition was represented by *n*. Data combinations were used for normalization and were computed as the sum of pairs of RFP measurements divided by the sum of pairs of GFP measurements for all pairwise combinations of the data. Measurements of GFP mRNA levels were used to normalize for transfection efficiency. The RFP/GFP ratio distribution did not exhibit normal or unimodal errors for the sample sizes used in this work. This was due to an observed γ distribution of GFP with a shape parameter less than 2. Hence, the distribution of the inverse of the GFP measurements had an extended tail. The use of data combinations reduced the extended tail distribution of 1/GFP, effectively generating a unimodal error distribution for the sample point averages.

The standardized mean of a contrast variable (SMCV) values were computed as method-of-moment estimates using Equation 1,

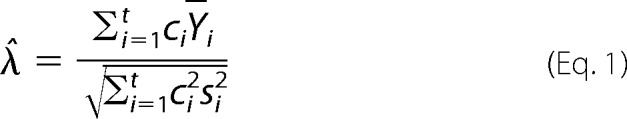
 where *t* represents the number of groups, *c_i_* is the contrast variable, *Ȳ_i_* is the group mean, and *s_i_* is the group variance (53). For cell type comparisons, SMCV values were computed on normalized data scaled between 0 and 100% within each cell type, where 100% was defined as the average maximum value attained by the undamaged control. The normalization had the effect of eliminating absolute differences among cell types so that curves could be directly compared. The SMCV values were converted to *c*^+^-probability values (53). The *c*^+^-probability values were converted to *p* values by using the relation,

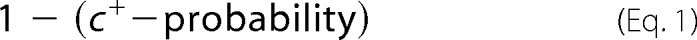
 which gives the probability of observing an SMCV value as extreme as the observed value under the null hypothesis that the SMCV value is ≤0. For the work described here, *p* values with an upper boundary of <0.050 are considered statistically significant.

##### Flow Cytometry

Flow cytometry and cell sorting were performed on at least three experimentally independent cell populations on a BD FACSAria^TM^ cell sorter (BD Biosciences). Photomultiplier tube voltages were chosen such that fluorescent cells remained within the dynamic range of the instrument without accumulating in the last channel. Excitation was achieved with a 488-nm laser. The cytometer settings in relevant collection channels were 290 V for the RFP photomultiplier tube using the PE-Texas-Red filter set (616/23 band pass filter) and 355 V for the GFP photomultiplier tube using the FITC filter set (530/30 band pass filter). Significantly fluorescent cells were separated based on a signal above autofluorescence in RFP compared with a channel that exhibited no specific fluorescence by using allophycocyanin (633 nm laser excitation, 670/30 band pass filter) and plotting GFP *versus* allophycocyanin. When processing data for fluorescence analysis, gates were drawn by hand in the program Cyflogic (CyFlo Ltd., Turku, Finland). RFP and GFP fluorescence values of gated cells were exported to a text file that was imported into the R programming environment. Custom scripts in R were written to perform fluorescence compensation to account for spectral overlap in RFP and GFP measurements (54). The value of log*_e_*(RFP/GFP) for each point was used to normalize RFP values (13). Following normalization, the induced cell population was determined using a mixture model to eliminate from consideration cells that did not respond to doxycycline (55).

## Results

### 

#### 

##### B[a]P-N^6^-dA Impedes hRNAPII Transcription Elongation

To investigate the role of DNA repair on abrogating the deleterious effects of DNA lesions that impede transcription, two adducts were selected, B[*c*]Ph-*N*^6^-dA and B[*a*]P-*N*^6^-dA. B[*c*]Ph-*N*^6^-dA is a poor substrate for global NER, but it poses a strong block to transcription, making it a likely substrate for TCR (56). In contrast, B[*a*]P-*N*^6^-dA, is an excellent substrate for global NER and might be an excellent substrate for TCR, but its effect on transcription had not yet been reported. To test the effect of a B[*a*]P-*N*^6^-dA adduct on RNA synthesis *in vitro*, a DNA template was generated that could support hRNAPII transcription. The template contained a single, site-specific B[*a*]P-*N*^6^-dA adduct positioned on the transcribed strand downstream from a CMV promoter; an analogous control template was made that lacked the damaged base, with dA located at the same site (Fig. 1*B*).

**FIGURE 3.**
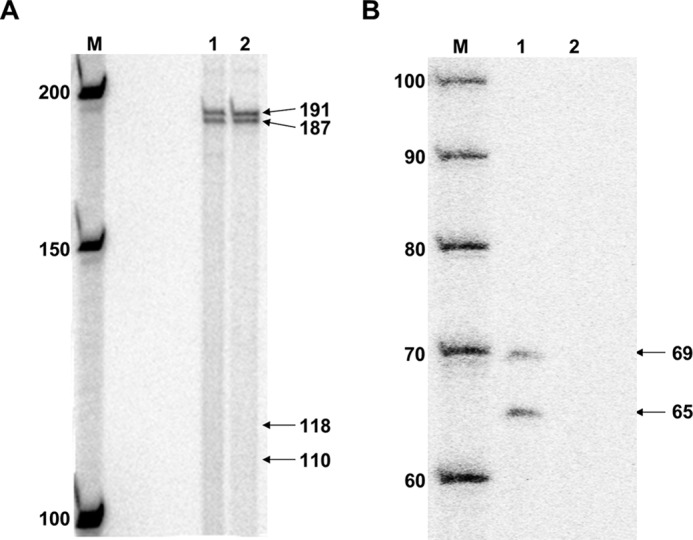
**DNA templates for *in vitro* transcription were characterized following synthesis.** *A*, I-PpoI digestion of DNA templates for *in vitro* transcription. *Lane M*, New England Biolabs 50-bp DNA ladder labeled with [^32^P]phosphate; *lane 1*, I-PpoI digestion of unmodified control DNA template; *lane 2*, I-PpoI digestion of B[*a*]P-*N*^6^-dA-modified DNA template. *B*, BsiWI digestion of DNA templates for *in vitro* transcription. *Lane M*, IDT 20/100 DNA ladder labeled with [^32^P]phosphate; *lane 1*, BsiWI digestion of unmodified control DNA template; *lane 2*, BsiWI digestion of B[*a*]P-*N*^6^-dA-modified DNA template.

Following template synthesis, it was essential to ensure that complete ligation had occurred; nicks in the template's backbone could impede transcription, confounding the results sought for the B[*a*]P-*N*^6^-dA adduct (57). To test for complete vector ligation, the templates were digested with the restriction endonuclease I-PpoI. The resulting oligomers were labeled with [^32^P]phosphate via an exchange reaction by incubating them with 83 μm [γ-^32^P]ATP (∼3 Ci/mol) and 100 μm ADP in the presence of T4 polynucleotide kinase. The products were resolved using 7% PAGE in the presence of 8 m urea. Complete template ligation should have resulted in oligomers that were 187, 191, 950, and 958 bases in length following I-PpoI digestion, whereas incomplete ligation should have resulted in additional oligomers 110 and 118 bases in length. As shown in Fig. 3*A*, bands in the vicinity of 110 and 118 bases were absent following I-PpoI digestion, both for the template modified with B[*a*]P-*N*^6^-dA and for unmodified control DNA. These results show that template ligation went to completion, at least within the assay's limits of detection, and that no nicks were present.

In addition to ensuring complete vector ligation, it was imperative to test for the presence of the B[*a*]P-*N*^6^-dA adduct following template synthesis. To do this, the site-specific B[*a*]P-*N*^6^-dA was positioned within a BsiWI restriction site, making the region insensitive to cutting by BsiWI relative to a control template lacking the adduct. Unmodified, control template or template containing B[*a*]P-*N*^6^-dA was digested with BsiWI. The resulting oligomers were labeled with [^32^P]phosphate and resolved using denaturing PAGE as described earlier. BsiWI digestion of the DNA template lacking the B[*a*]P-*N*^6^-dA adduct should result in three DNA fragments 65, 69, and 1076 bases in length following denaturation. Bands ∼65 and 69 bases in length were present following digestion of the unmodified, control DNA as predicted (Fig. 3*B*, *lane 1*). Note that the larger bands are not shown on the gel. In contrast, BsiWI digestion of the DNA template containing a site-specific B[*a*]P-*N*^6^-dA adduct within the BsiWI restriction site resulted in no DNA fragments that were 65 and 69 bases in length, indicating that the adduct was present following template synthesis (Fig. 3*B*, *lane 2*).

**FIGURE 4.**
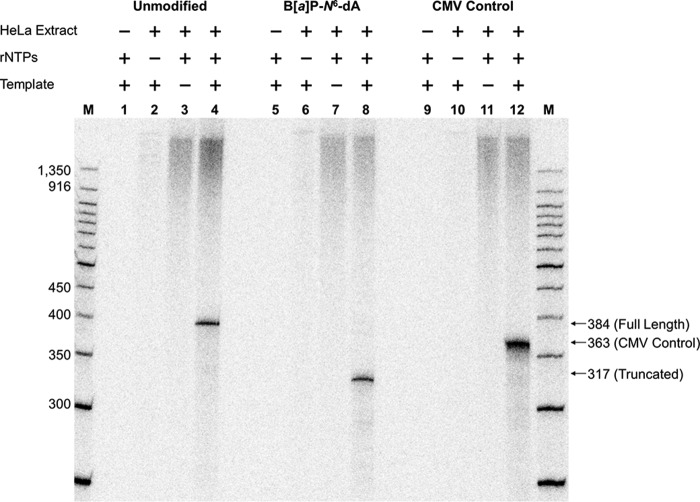
**B[*a*]P-*N*^6^-dA blocks hRNAPII elongation.** For each DNA template studied, four separate reactions were carried out as indicated above each lane in the gel: *lane M*, New England Biolabs 50-bp DNA ladder labeled with [^32^P]phosphate; *lanes 1–4*, results for transcription using the unmodified DNA template illustrated in Fig. 1; *lanes 5–8*, transcription results using the DNA template modified with a B[*a*]P-*N*^6^-dA adduct, also illustrated in Fig. 1; *lanes 9–12*, results using the control DNA template containing a CMV immediate early promoter supplied with the HeLa nuclear extract. The components for each transcription reaction are indicated *above* the *lanes*.

After template integrity was confirmed, transcription was examined *in vitro* using HeLa nuclear extract as the source of hRNAPII. Reactions were carried out for 1 h at 30 °C using [α-^32^P]CTP among the nucleotides needed for RNA synthesis. The HeLa nuclear extract was tested for activity by using a DNA template supplied by the manufacturer, which encodes a transcript 363 bases in length. Production of RNA approximating this length was observed only when the manufacturer's template was incubated with HeLa nuclear extract and rNTPs, demonstrating that the extract was indeed active (Fig. 4, *lanes 9–12*).

*In vitro* transcription using undamaged, control DNA template, should result in the production of full-length, run-off RNA that is 384 bases in length when hRNAPII initiates at the +1 start site associated with the CMV promoter. In contrast, transcription using the DNA template with a B[*a*]P-*N*^6^-dA lesion positioned at the +317 site should result in RNA ∼317 bases in length if the adduct were to impede elongation. Transcription of the control template in which all necessary components of the reaction were present resulted in the production of RNA 380 bases in length, which approximates the length of expected run-off transcription (Fig. 4, *lane 4*). Reactions in which one critical component was absent (HeLa extract, NTPs, or template DNA) resulted in no visible RNA approximating 380 bases in length (Fig. 4, *lanes 1–3*). These results indicate that the synthesized template lacking B[*a*]P-*N*^6^-dA supports transcription by hRNAPII, resulting in a run-off transcript of the expected size. Note that synthesis of the larger transcripts in lanes with HeLa nuclear extract is a function of the extract itself.

In contrast to the results obtained for the unmodified control template, transcription of the template containing a site-specifically modified B[*a*]P-*N*^6^-dA adduct produced a single band of ∼320 bases, significantly smaller than that produced from the unmodified template and approximating the 317-base RNA that would result from hRNAPII stalling at B[*a*]P-*N*^6^-dA (Fig. 4, *lane 8*). Note again that reactions lacking one critical component produced no RNA in the vicinity of 320 or 380 bases (Fig. 4, *lanes 5–7*). These results clearly indicate that the B[*a*]P-*N*^6^-dA adduct poses a strong block to hRNAPII during elongation, suggesting that this lesion should be subject to TCR in cells.

##### Cellular DNA Repair Phenotypes Influence the Expression of Genes That Contain either B[a]P-N^6^-dA or B[c]Ph-N^6^-dA in the Transcription Unit

To test the effect of DNA repair on RNA synthesis from genes containing B[*a*]P-*N*^6^-dA or B[*c*]Ph-*N*^6^-dA, primary human fibroblasts were transfected with site-specifically damaged vectors that lacked identifiable eukaryotic origins of replication (13). The vector contained two reporter genes: a constitutively active GFP gene and an RFP gene under the control of a tetracycline-inducible promoter (58). A site-specific B[*a*]P-*N*^6^-dA or a B[*c*]Ph-*N*^6^-dA adduct was positioned on the transcribed strand of the RFP gene within the 5′-UTR (Fig. 2). In essence, the production of GFP mRNA and GFP was used to monitor transfection, and production of RFP mRNA and RFP was used to assess the effect of the lesion on transcription and subsequent translation in primary human fibroblasts. Importantly, the cells studied had different DNA repair backgrounds as follows: normal human fibroblasts (GM03651) (59), XPA^−/−^ human fibroblasts (GM05509) (60), XPC^−/−^ human fibroblasts (GM02993) (61), and CSB^−/−^ human fibroblasts (GM01629) (62) (Coriell Cell Repositories).

**FIGURE 5.**
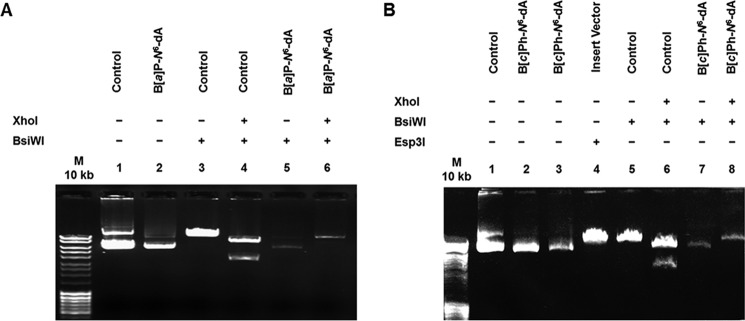
**Site-specifically modified vectors for transcription in cells were characterized.** The presence of either B[*a*]P-*N*^6^-dA (*A*) or B[*c*]Ph-*N*^6^-dA adducts (*B*) within a BsiWI restriction sight located in the vector prevents BsiWI digestion. Vectors were incubated with the restriction enzymes shown, and the products were resolved by agarose gel electrophoresis.

Prior to transfection experiments, the integrity of the DNA constructs was examined to ensure that the DNA adduct was intact following vector synthesis. Each of the three vectors (unmodified control DNA, DNA modified with B[*a*]P-*N*^6^-dA, and DNA modified with B[*c*]Ph-*N*^6^-dA) contained a single XhoI restriction site and a single BsiWI restriction site. The B[*a*]P-*N*^6^-dA adduct and the B[*c*]Ph-*N*^6^-dA adduct were positioned within the BsiWI site, making it refractory to cutting by the BsiWI restriction endonuclease. Hence, each vector should be sensitive to cutting with XhoI, but only the unmodified control vector should be sensitive to cutting with BsiWI as well. Each of the three vectors was cut with XhoI and BsiWI or with BsiWI alone. The results are presented in Fig. 5.

Characterization of the DNA construct containing the B[*a*]P-*N*^6^-dA is shown in Fig. 5*A*. The uncut control DNA and uncut DNA containing the B[*a*]P-*N*^6^-dA are shown in *lanes 1* and *2*, respectively. Control DNA and the B[*a*]P-*N*^6^-dA vector cut with BsiWI alone are shown in *lanes 3* and *5*, respectively. As predicted, only the control vector was sensitive to cutting by BsiWI. Control DNA and the B[*a*]P-*N*^6^-dA vector cut with both XhoI and BsiWI are shown in *lanes 4* and *6*, respectively. Only the control vector was sensitive to cutting by both enzymes, resulting in two bands; the B[*a*]P-*N*^6^-dA vector was cut only once, resulting in a shift from a closed circular form to a linear form. These results indicate that the B[*a*]P-*N*^6^-dA adduct was present in the BsiWI site.

Characterization of the DNA construct containing the B[*c*]Ph-*N*^6^-dA DNA is shown in Fig. 5*B*. The uncut control DNA is shown in *lane 1*, and uncut DNA containing the B[*c*]Ph-*N*^6^-dA is shown in *lanes 2* and *3*. Control DNA and the B[*c*]Ph-*N*^6^-dA vector cut with BsiWI alone are shown in *lanes 5* and *7*, respectively. Again, as predicted, only the control vector was sensitive to cutting by BsiWI. Control DNA and the B[*c*]Ph-*N*^6^-dA vector cut with both XhoI and BsiWI are shown in *lanes 6* and *8*, respectively. Only the control vector was sensitive to cutting by both enzymes, resulting in two bands; the B[*c*]Ph-*N*^6^-dA vector was cut only once, as expected. These results indicate that the B[*c*]P-*N*^6^-dA adduct was present in the BsiWI site in this construct. (Note that *lane 4* shows the insert vector cut with Esp3I, which cuts twice, resulting in two pieces of DNA, a long fragment 11,475 bp in length and a very short fragment 73 bp in length that does not appear on the gel).

**FIGURE 6.**
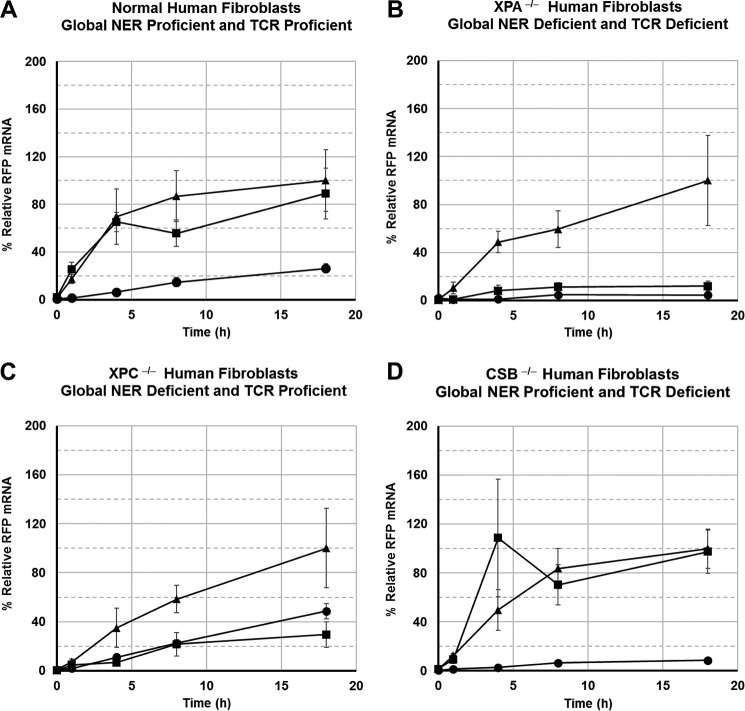
**B[*c*]Ph-*N*^6^-dA interferes with transcription elongation in all cells lacking NER or TCR, whereas B[*a*]P-*N*^6^-dA only exhibits an effect when NER is compromised.** *A*, normal primary human fibroblasts; *B*, XPA^−/−^ primary human fibroblasts; *C*, XPC^−/−^ primary human fibroblasts; *D*, CSB^−/−^ primary human fibroblasts. Time courses for each cell type: unmodified control vector (▴); vector modified with B[*a*]P-*N*^6^-dA (■); vector modified with B[*c*]Ph-*N*^6^-dA (●). RFP mRNA levels were normalized as the percentage of the maximum average RFP mRNA level compared with that of the control vector in a given cell type.

After the presence of the adducts was confirmed for each vector, cells were transfected with damaged vector or corresponding undamaged control vector and grown in minimal essential medium supplemented with 15% FBS. After 24 h, doxycycline was added to stimulate transcription of the RFP gene. Cells were harvested at 0, 1, 4, 8, and 18 h following induction of transcription, and total RNA was isolated. GFP mRNA and RFP mRNA were measured using quantitative RT-PCR at each time point. Normalized RFP mRNA quantities for cells transfected with vectors containing B[*a*]P-*N*^6^-dA and B[*c*]Ph-*N*^6^-dA lesions were plotted as a function of time for each cell line (Fig. 6).

In DNA repair-proficient normal human fibroblasts, the B[*a*]P-*N*^6^-dA adduct exerted no significant effect on the quantity of mRNA obtained from transcription of the RFP gene when compared with mRNA obtained from an undamaged control vector (*p* < 0.5). However, the B[*c*]Ph-*N*^6^-dA adduct exerted a significantly adverse effect on transcription of the RFP gene in human cells proficient in DNA repair when compared with transcription of the unmodified, control vector (*p* < 0.02) (Fig. 6*A*). Furthermore, the B[*a*]P-*N*^6^-dA and B[*c*]Ph-*N*^6^-dA transcription time courses were also significantly different from one another in normal human fibroblasts, with less RFP mRNA resulting from the vector containing B[*c*]Ph-*N*^6^-dA when compared with B[*a*]P-*N*^6^-dA (*p* < 0.01). These results suggest that the B[*a*]P-*N*^6^-dA adduct is rapidly removed from DNA or bypassed during transcription in cells that are proficient in DNA repair. In contrast, the detrimental effects of the B[*c*]Ph-*N*^6^-dA adduct on transcription persist in normal cells, interfering with mRNA synthesis for at least 18 h following activation of gene expression in cells that are proficient in both global NER and TCR.

In primary human fibroblasts derived from patients with XP, complementation group A, that are deficient in both global NER and TCR, both B[*a*]P-*N*^6^-dA and B[*c*]Ph-*N*^6^-dA exert severe, adverse effects on the production of RFP mRNA when compared with an undamaged control vector (*p* < 0.03 and *p* < 0.02, respectively) (Fig. 6*B*). The quantity of RFP mRNA obtained from a vector containing B[*c*]Ph-*N*^6^-dA appeared to be slightly lower than that obtained from a vector containing B[*a*]P-*N*^6^-dA, although the difference was not statistically significant (*p* < 0.06). These data suggest that the XPA protein, which is involved in both global NER and TCR, is necessary to ameliorate the negative effects of both B[*a*]P-*N*^6^-dA and B[*c*]Ph-*N*^6^-dA on transcription. The data also show that a small amount of full-length mRNA is made via transcription of the template even in the absence of global NER and TCR.

In primary human fibroblasts derived from patients with XP, complementation group C, that are defective in global NER but proficient in TCR, both B[*a*]P-*N*^6^-dA and B[*c*]Ph-*N*^6^-dA exert a significantly negative effect on the production of RFP mRNA compared with an undamaged control vector (*p* < 0.04 and *p* < 0.05, respectively) (Fig. 6*C*). Importantly, there was no significant difference between the effects of B[*a*]P-*N*^6^-dA and B[*c*]Ph-*N*^6^-dA on RNA synthesis when the two damaged vectors were compared with one another (*p* < 0.4). For the B[*c*]Ph-*N*^6^-dA lesion, the RFP mRNA increases to levels comparable with that in normal cells, but the overall deleterious effect is still significant. These data indicate that the XPC protein, which is absent from these cells, assists significantly in negating the detrimental effects of B[*a*]P-*N*^6^-dA on transcription but has a nominal impact on the effect of B[*c*]Ph-*N*^6^-dA.

In primary human fibroblasts derived from patients with CS, complementation group B, that are defective in TCR but proficient in global NER, the B[*a*]P-*N*^6^-dA adduct had no significant effect on transcription (*p* < 0.5), whereas the B[*c*]Ph-*N*^6^-dA adduct impeded transcription to a very large and significant extent (*p* < 0.01) (Fig. 6*D*). The transcript levels produced from vectors containing either B[*a*]P-*N*^6^-dA or B[*c*]Ph-*N*^6^-dA were also significantly different from one another (*p* < 0.01).

An important question emerges. Is TCR operating on B[*a*]P-*N*^6^-dA and B[*c*]Ph-*N*^6^-dA to reactivate the RFP gene? If TCR were acting on the B[*c*]Ph-*N*^6^-dA lesion, there should be significantly better recovery of RFP gene expression in normal human fibroblasts or XPC^−/−^ cells in which TCR operates when compared with XPA^−/−^ or CSB^−/−^ cells in which TCR is defective. Comparisons for transcription of the vector containing a B[*c*]Ph-*N*^6^-dA lesion show that RFP mRNA quantities were significantly higher in XPC^−/−^ cells compared with XPA^−/−^ cells (*p* < 0.01) or compared with CSB^−/−^ cells (*p* < 0.01). These comparisons show that TCR operates on B[*c*]Ph-*N*^6^-dA to a significant extent, a conclusion that is also borne out by the RFP that is made as shown below. In contrast, it is less clear that TCR operates efficiently on B[*a*]P-*N*^6^-dA. For RFP mRNA recovery from the vector containing B[*a*]P-*N*^6^-dA, XPC^−/−^ cells were not significantly better at reactivating gene expression when compared with XPA^−/−^ cells (*p* < 0.1). Although the results were not statistically significant by SMCV analysis, the quantity of RFP mRNA generated relative to the control at 18 h was clearly higher in XPC^−/−^ cells (29.5 ± 10.4%) than in XPA^−/−^ cells (12.0 ± 3.9%). This supports the notion that TCR does operate on the B[*a*]P-*N*^6^-dA adduct, but significantly observable differences are seen only at later time points.

##### Recovery of Active RFP from an RFP Gene Containing B[a]P-N^6^-dA or B[c]Ph-N^6^-dA Is Dependent on the Cell's DNA Repair Phenotype

Detection of full-length RFP mRNA in the transfection experiments suggests that RFP should be made even in cells in which the quantity of RFP mRNA was quite low. To test this, primary human fibroblasts of known DNA repair status were transfected with unmodified control vectors or those containing a B[*a*]P-*N*^6^-dA or B[*c*]Ph-*N*^6^-dA adduct within the 5′-UTR of the RFP gene's transcription unit. RFP activity following transfection was measured using FACS.

**FIGURE 7.**
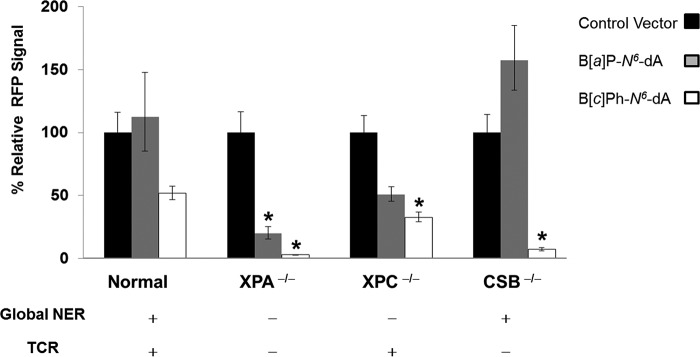
**The effect of DNA repair background on recovery of RFP from vectors containing either B[*a*]P-*N*^6^-dA or B[*c*]Ph-*N*^6^-dA.** Each *cluster* of *three bars* represents the mean and S.E. (*error bars*) of the mean of the normalized RFP signal for the indicated cell type, with a value of 100% for the RFP signal for the control. Shown are control vector (*black*), vector modified with B[*a*]P-*N*^6^-dA (*gray*), and vector modified B[*c*]Ph-*N*^6^-dA (*white*). *, statistically significant difference from the respective control (*p* < 0.05). Statistical tests were performed by one-way analysis of variance with contrasts.

In normal human fibroblasts proficient in both global NER and TCR, the RFP signal generated from a vector containing a B[*a*]P-*N*^6^-dA lesion was not significantly different from the RFP signal from a control vector (*p* = 0.99), consistent with the RFP mRNA data (Fig. 7). In contrast, the mean RFP signal resulting from transfection of normal human fibroblasts with a vector containing a B[*c*]Ph-*N*^6^-dA lesion was lower than that of the corresponding control (52% of the control value), also consistent with the RFP mRNA data, but the difference was not statistically significant for these data (*p* = 0.41). These results indicate that the B[*c*]Ph-*N*^6^-dA adduct is less well tolerated than B[*a*]P-*N*^6^-dA, even when global NER and TCR are operative.

In XPA^−/−^ cells deficient in both global NER and TCR, the RFP signals generated from vectors containing either a B[*a*]P-*N*^6^-dA or B[*c*]Ph-*N*^6^-dA adduct were significantly lower than RFP from a control vector (*p* = 0.000036 and *p* = 3.6 × 10^−12^, respectively). These results indicate that the XPA protein is important in abrogating the impact of each of these adducts on transcription, more than likely by assisting in their removal via global NER and TCR. Interestingly, the RFP signal from a B[*c*]Ph-*N*^6^-dA vector was significantly lower than that from the B[*a*]P-*N*^6^-dA vector (*p* = 0.00011), suggesting that B[*a*]P-*N*^6^-dA is either more effectively bypassed by hRNAPII than B[*c*]Ph-*N*^6^-dA or potentially that B[*a*]P-*N*^6^-dA is slowly processed by additional repair pathways that do not require the XPA protein.

In XPC^−/−^ cells, which lack global NER but are proficient in TCR, the RFP signals obtained from vectors containing either a B[*a*]P-*N*^6^-dA or B[*c*]Ph-*N*^6^-dA adduct were both lower than the RFP signal obtained from the control vector (51 and 33% of the control value, respectively); however, only the B[*c*]Ph-*N*^6^-dA adduct produced results of statistical significance when compared with the control (*p* = 0.023).

In CSB^−/−^ cells that lack TCR, there is no significant effect of a B[*a*]P-*N*^6^-dA adduct on RFP fluorescence. In marked contrast, very little RFP signal results following transfection with a vector containing B[*c*]Ph-*N*^6^-dA (*p* = 1.2 × 10^−8^). In addition, the RFP signal resulting from the B[*c*]Ph-*N*^6^-dA-modified vector was significantly lower than that obtained from the B[*a*]P-*N*^6^-dA-modified vector. This suggests that the CSB protein is involved in diminishing the effects of B[*c*]Ph-*N*^6^-dA on transcription, and the effect is far more pronounced than that for B[*a*]P-*N*^6^-dA.

To consider further the effect of TCR on recovery of transcription from templates with a B[*a*]P-*N*^6^-dA or B[*c*]Ph-*N*^6^-dA lesion, the differences in RFP fluorescence between cell types were elucidated. Note that protein recovery followed a similar pattern to that for RFP mRNA expression.

For the vector containing B[*c*]Ph-*N*^6^-dA, significantly higher RFP recovery was observed in normal fibroblasts compared with XPA^−/−^ cells that are deficient in global NER and TCR (*p* = 4.2 × 10^−6^) and CSB^−/−^ cells that are only deficient in TCR (*p* = 0.0014). Indeed, RFP levels were greater in XPC^−/−^ cells than in XPA^−/−^ cells (*p* = 0.00010) and CSB^−/−^ cells (*p* = 0.030), a reflection of the ability of XPC^−/−^ cells to carry out TCR that is compromised in both XPA^−/−^ cells and CSB^−/−^ cells.

For the vector containing B[*a*]P-*N*^6^-dA, significantly better recovery of RFP mRNA and RFP would be expected in XPC^−/−^ cells in which TCR could operate on the lesion, especially in comparison with recovery in XPA^−/−^ cells in which both NER and TCR are absent. Indeed, RFP fluorescence from cells transfected with the vector containing B[*a*]P-*N*^6^-dA was 2.6-fold higher in XPC^−/−^ cells relative to XPA^−/−^ cells, but the difference was not statistically significant (*p* = 0.34). However, the observed trend for these data is in the expected direction, again suggesting that repair pathways other than NER operate on B[*a*]P-*N*^6^-dA. This is in sharp contrast to recovery of RNA synthesis from a transcription unit containing B[*c*]Ph-*N*^6^-dA in which TCR appears to be the principal facilitator of its removal.

## Discussion

DNA damage located within a gene's transcription unit often delays or obstructs the progression of RNA polymerase, a potentially dire situation for the precise temporal and spatial regulation needed for effective gene expression. However, a stalled RNA polymerase can in turn act as a signal for TCR (9, 13, 63). Indeed, TCR may well have evolved precisely to ensure that interruptions to transcription by DNA damage are kept at bay (64).

In this study, the *in vitro* and *in vivo* effects on transcription of two topologically distinct DNA adducts, B[*a*]P-*N*^6^-dA and B[*c*]Ph-*N*^6^-dA, were examined using cell extracts and primary human fibroblasts. B[*a*]P-*N*^6^-dA poses a strong block to hRNAPII progression *in vitro* and is an excellent substrate for NER *in vitro* (44, 65). In contrast, B[*c*]Ph-*N*^6^-dA, which was previously shown to act as a potent block to hRNAPII transcription *in vitro* (56), is a relatively poor substrate for NER *in vitro* (34). The results reported here show that both B[*a*]P-*N*^6^-dA and B[*c*]Ph-*N*^6^-dA exert a profound, negative effect on transcription in human cells that lack both global NER and TCR, results that align well with *in vitro* data showing that both adducts strongly impede transcription elongation and RNA synthesis. Furthermore, and as predicted, the challenge to transcription elongation that both adducts pose is less severe in cells that execute TCR and global NER. However, the overall deleterious effect on transcription is more pronounced for B[*c*]Ph-*N*^6^-dA than for B[*a*]P-*N*^6^-dA, because the latter is subject to repair by global NER, and the former is resistant to repair by that pathway. TCR operates on B[*c*]Ph-*N*^6^-dA and perhaps on B[*a*]P-*N*^6^-dA as well. For B[*a*]P-*N*^6^-dA, cells proficient in global NER and deficient in TCR reactivate the gene in which the adduct is present to levels equivalent to those of an undamaged gene, a result that is consistent with global NER repairing the damage. In XPC^−/−^ cells that lack NER but exhibit active TCR, reactivation of the gene occurs as well, but the quantity of transcripts does not return to the same level as that observed for transcription of the undamaged gene within the time frame investigated. Hence, B[*a*]P-*N*^6^-dA represents an example of a DNA adduct that is repaired by global NER and quite possibly by TCR as well. Indeed, in cells proficient in global NER, repair of the B[*a*]P-*N*^6^-dA adduct may well occur during the 24-h period prior to activation of the RFP gene. This is consistent with the rapid recovery of both RFP mRNA and RFP activity observed in human fibroblasts that are proficient in global NER but that lack TCR. In contrast, the B[*c*]Ph-*N^6^*-dA lesion limits transcript production even further in cells that lack TCR but are proficient in global NER, an observation that is consistent with the adduct's ability to escape the latter pathway. Recently, a host cell reactivation approach was employed to demonstrate that yet another lesion, a single 3-(deoxyguanosin-*N*^2^-yl)-2-acetylaminofluorene adduct, also escapes global NER and requires TCR for repair in SV40 transformed skin fibroblasts (66). These results are consistent with the results reported here in untransformed human fibroblasts using different bulky DNA lesions.

In addition to its role in the recovery of gene expression following damage to the genome, DNA repair protects the integrity of the resulting transcripts by minimizing transcriptional mutagenesis. Qualitative, preliminary data from deep sequencing of transcripts recovered from the cell lines used in this study show that the full-length transcripts resulting from expression of the site-specifically modified RFP gene were mostly normal in human fibroblasts that are proficient in global NER and TCR, particularly for the adduct B[*a*]P-*N*^6^-dA, where no altered transcripts were observed. However, in cells lacking functional XPA protein, which makes them deficient in NER, both globally and via TCR, a relatively small number of full-length transcripts was observed, but the mRNA contained significant base misincorporations, with adenosine or cytidine inserted at the position opposite B[*a*]P-*N*^6^-dA or adenosine and guanosine or cytidine inserted at the position opposite B[*c*]Ph-*N*^6^-dA. In addition deletions were seen. Indeed, base misincorporations and deletions in mRNA were also observed in other cell lines deficient in DNA repair for transcription of vectors containing B[*a*]P-*N*^6^-dA or B[*c*]Ph-*N*^6^-dA (Table 3).

**TABLE 3:**
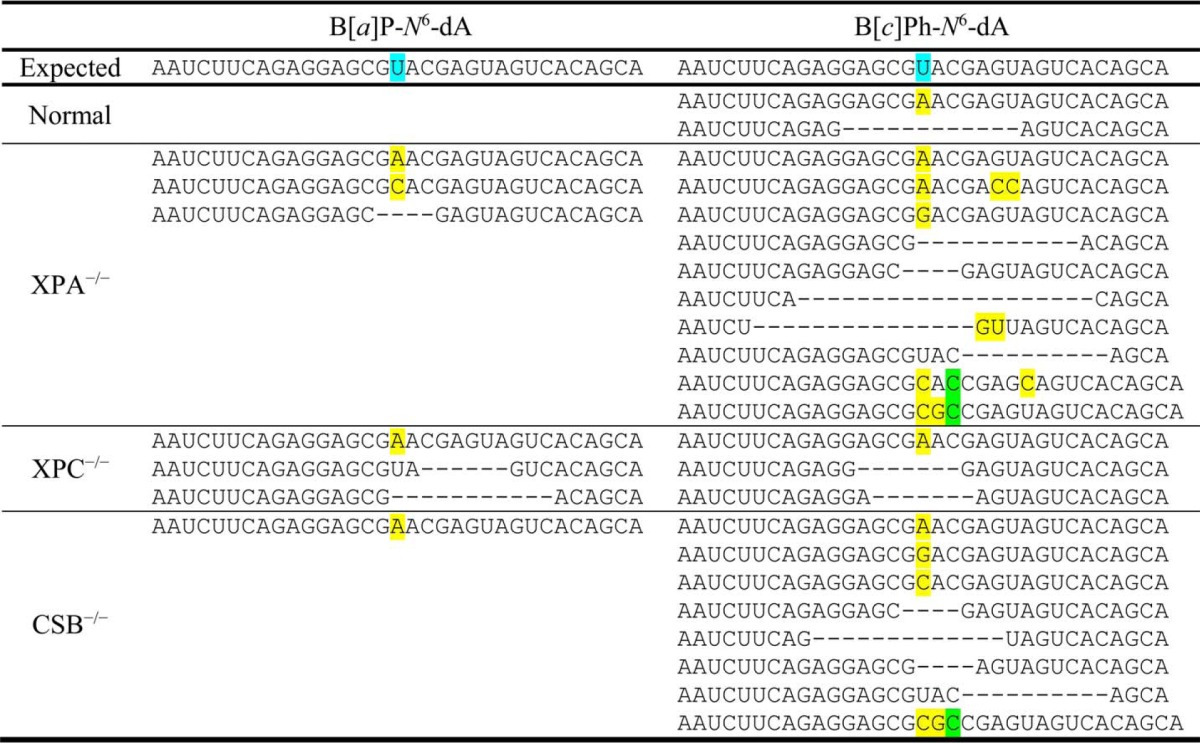
**Representative base misincorporations, deletions and insertions observed in mRNA following transcription past B[*a*]P-*N*^6^-dA or B[*c*]Ph-*N*^6^-dA** The mRNA data shown were generated by amplifying cDNA with barcoded primers containing Illumina HiSeq adapters, and sequencing the resulting amplicons on a HiSeq 2500 System (Illumina, San Diego, CA). Analysis of the resultant reads was accomplished with custom Perl scripts. The mRNA shown represent accumulated, abnormal transcripts that were observed at a frequency ≥10 times the background misincorporation rate of 0.1% in each sample. Only the portions of the full-length transcripts in the vicinity of the DNA adduct are shown. Bases highlighted in yellow indicate sites of misincorporation; bases highlighted in green indicate insertions; and a dash indicates the site of deletions.(Additional experiments are needed to understand how the pattern of abnormal transcripts changes quantitatively over time.) Normal cells are proficient in both global NER and TCR; XPA^−/−^ cells are deficient in both global NER and TCR; XPC^−/−^ cells are deficient in global NER but proficient in TCR; and CSB^−/−^ cells are proficient in global NER but deficient in TCR. The expected mRNA sequence is shown, with the U marked in blue indicating the complementary position within the DNA template's transcription unit that was modified with either B[*a*]P-*N*^6^-dA or B[*c*]Ph-*N*^6^-dA. Note that in normal, DNA repair-proficient fibroblasts, no abnormal transcripts were observed; in all other fibroblasts, normal mRNA was found along with the abnormal transcripts.

A plausible explanation for the generation of full-length transcripts is slow, error-free, or erroneous bypass of the lesion by hRNAPII, as previously documented in the case of cyclobutane pyrimidine dimers induced by ultraviolet radiation and oxidative DNA damage (67–69). In fact, *in vitro* transcription data show that some bypass of B[*c*]Ph-*N*^6^-dA by hRNAPII does occur (56). Alternatively, spontaneous depurination at the damaged site with subsequent DNA repair could result in the full-length transcripts observed that do not contain base misincorporations or deletions (70). Another possibility is the involvement of an alternate repair pathway, such as homologous recombination (71), mismatch repair (72), or nucleotide incision repair (73). Finally, acquisition of nucleosome-like structures, which plasmids adopt after transfection, could influence the expression of its associated genes and their susceptibility to DNA repair (74–76).

**FIGURE 8.**
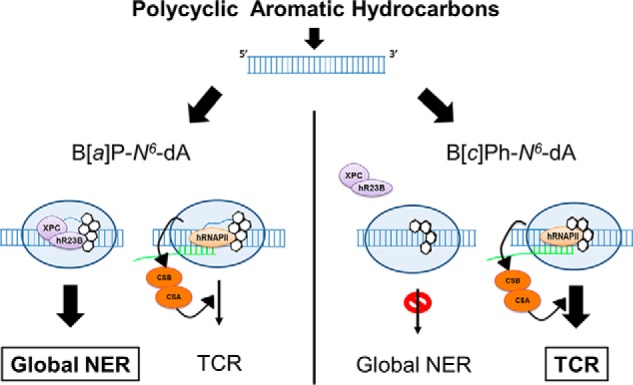
**B[*c*]Ph-*N*^6^-dA, which is resistant to NER, is subject to TCR; B[*a*]P-*N*^6^-dA is subject to global NER, with the strong possibility that other repair pathways, including TCR, play a role in its removal.** NER repairs B[*a*]P-*N*^6^-dA efficiently, permitting reactivation of gene expression. In the absence of global NER, B[*a*]P-*N*^6^-dA is repaired by other pathways, more than likely including TCR. In contrast, B[*c*]Ph-*N*^6^-dA is a very poor substrate for global NER, with TCR mediating its repair to reactivate gene expression.

In Fig. 8, a model is presented to explain the differential effects of B[*a*]P-*N*^6^-dA and B[*c*]Ph-*N*^6^-dA on transcription in cells with varying DNA repair phenotypes. B[*a*]P-*N*^6^-dA is accessible to global NER, making TCR less relevant for its removal. In contrast, the B[*c*]Ph-*N*^6^-dA adduct adopts a conformation that allows it to escape detection by global NER, resulting in its persistence within the genome. However, when B[*c*]Ph-*N*^6^-dA is located on the transcribed strand within an expressed transcription unit, its ability to block RNA polymerase elongation results in TCR acting on the adduct. These results are consistent with the relatively high tumorigenic potential observed for B[*c*]Ph relative to B[*a*]P, since some adducts formed by the former PAH are more persistent in the genome.

The results reported here show that TCR ameliorates the deleterious effect of DNA adducts that block transcription, presumably by enhancing their removal from active genes even when they escape repair by global NER and perhaps other DNA pathways as well. Furthermore, the absence of global NER, TCR, or both results in decreased gene expression and alterations to the sequence of the mRNA produced. Hence, TCR protects transcription units from the dire consequences of transcription-blocking DNA adducts by reactivating gene expression and protecting the integrity of the resulting transcripts, including lesions that might be resistant to repair via other pathways.

## Author Contributions

A. N., J. A. B., and D. A. S. designed the study and wrote the paper. A. G. collaborated on the statistical analysis of the data. M. A. C. carried out *in vitro* transcription reactions. L. C. prepared vector DNA for the studies in cells. C. B. supplied parent plasmids and collaborated on the design of the vectors used in this research. N. E. G. synthesized and contributed the site-specifically modified DNA. All authors approved the final version of the manuscript.

## Footnotes

3 The abbreviations used are:

NER: nucleotide excision repair
TCR: transcription-coupled repair
XP: xeroderma pigmentosum
CS: Cockayne syndrome
PAH: polycyclic aromatic hydrocarbons
B[*a*]P: benzo[*a*]pyrene
B[*c*]Ph: benzo[*c*]phenanthrene
B[*a*]P-*N*^6^-dA: (+)*-trans-anti*-B[*a*]P-*N*^6^-dA
B[*c*]Ph-*N*^6^-dA: (+)*-trans-anti*-B[*c*]Ph-*N*^6^-dA
hRNAPII: human RNA polymerase II
qPCR: quantitative polymerase chain reaction
SMCV: standardized mean of a contrast variable.
